# Inflammation induced by increased frequency of intermittent hypoxia is attenuated by tempol administration

**DOI:** 10.1590/1414-431X20154487

**Published:** 2015-09-18

**Authors:** J. Zhang, L. Zheng, J. Cao, B. Chen, D. Jin

**Affiliations:** Department of Respiratory, Tianjin Medical University General Hospital, Tianjin, China

**Keywords:** Obstructive sleep apnea, Intermittent hypoxia, Inflammation, Nuclear factor kappa B, Antioxidant

## Abstract

The levels of serum inflammatory cytokines and the activation of nuclear factor kappa B (NF-κB) and hypoxia inducible factor-1α (HIF-1α) in heart tissues in response to different frequencies of intermittent hypoxia (IH) and the antioxidant tempol were evaluated. Wistar rats (64 males, 200-220 g) were randomly divided into 6 experimental groups and 2 control groups. Four groups were exposed to IH 10, 20, 30, or 40 times/h. The other 2 experimental groups were challenged with IH (30 times/h) plus tempol, either beginning on day 0 (IH30T0) or on day 29 (IH30T29). After 6 weeks of challenge, serum levels of tumor necrosis factor (TNF)-α, intracellular adhesion molecule (ICAM)-1, and interleukin-10 were measured, and western blot analysis was used to detect NF-κB p65 and HIF-1α in myocardial tissues. Serum levels of TNF-α and ICAM-1 and myocardial expression of NF-κB p65 and HIF-1α were all significantly higher in IH rats than in controls (P<0.001). Increased IH frequency resulted in more significant changes. Administration of tempol in IH rats significantly reduced levels of TNF-α, ICAM-1, NF-κB and HIF-1α compared with the non-tempol-treated group (F=16.936, P<0.001). IH induced an inflammatory response in a frequency-dependent manner. Additionally, HIF-1α and NF-κB were increased following IH administration. Importantly, tempol treatment attenuated this effect.

## Introduction

Sleep apnea is a common medical condition, primarily caused by complete or partial pharyngeal obstruction and characterized by the repeated cessation of breathing while asleep. The disorder is commonly accompanied by hypoxia, sleep arousal, and hemodynamic changes (1). Persistent recurrence of these conditions can lead to a number of deleterious effects, ranging from sleepiness, fatigue, and poor neurocognitive performance during the day to more severe symptoms, including cardiovascular disease, cerebrovascular disease, and multiple organ injury. Obstructive sleep apnea (OSA) is the most common form of the disorder, and intermittent hypoxia (IH; intermittent periods of oxygen saturation below 90%) is a key driver of the pathophysiology of this disease. The harmful effects of IH have been compared with those resulting from ischemia-reperfusion injury, and are thought to be caused by increased endothelial cell dysfunction and inflammation. Importantly, OSA is considered a chronic low-grade inflammatory disease (2,3) and a growing body of evidence links OSA with the development and progression of cardiovascular disease (CAD). Treatment of OSA with continuous positive airway pressure in symptomatic patients without known CAD has been shown to decrease morbidity and mortality associated with cardiovascular events (4). The pathophysiology underlying the association between OSA and CAD is not fully established, but several mechanisms have been proposed, including hyperactive sympathetic drive, vascular inflammation, oxidative stress, endothelial damage, and metabolic deregulation (5).

Previous studies have shown that IH during sleep results in the increased production of reactive oxygen species (ROS) in response to oxidative stress activation. ROS, including superoxide anion (O_2_^•−^), hydroxyl radical (OH^−^), and peroxynitrite (ONOO^−^), are second messengers capable of activating and regulating nuclear factor kappa B (NF-κB), hypoxia inducible factor-1 alpha (HIF-1α), and activator protein-1 (AP-1), transcription factors that regulate genes involved in inflammation and adhesion (6). Although these events set into motion an inflammatory cascade, it is unclear whether the frequency of IH influences the activation and release of inflammatory and adhesive factors.

## Material and Methods

### Animals

A total of 64 male Wistar rats (approximately 8 weeks of age, 200-220 g), acquired from the animal experimental center of the Military Medical Science Academy, Tianjin, China, were used in this study. Rats were housed 4 per cage. At all times except during the actual experimental period, rats were provided food and water *ad libitum*. All surgical procedures and experimental protocols were approved by the Tianjin Medical University Animal Care and Use Committee.

### Intermittent hypoxia challenge

Rats were randomly assigned to 8 groups containing 8 rats per group. Rats in 4 groups received IH 10 (IH10), 20 (IH20), 30 (IH30), or 40 (IH40) times per hour, respectively. Tempol (STBB3145, Sigma, USA) was freshly prepared each day and was administered by intraperitoneal injection at 100 mg/kg before each IH challenge (30 times/h) in 2 groups of rats on the first day of challenge until the end of the experimental period (IH30T0) or beginning on day 29 of the procedure (IH30T29). Rats in the last 2 groups received compressed air (SC), or were maintained under normal air conditions (NC).

For IH exposure, rats were placed in a specialized Plexiglas chamber (30×20×20 cm^3^, with 4 per cage) and were exposed to IH for 8 h/day (9:00 am to 5:00 pm) for 7 days/week for 6 consecutive weeks. The chamber was flushed with alternating cycles of pure nitrogen and compressed air. Cycles of IH lasted for 6, 3, 2, and 1.5 min. The first 30 s of each cycle was defined as the hypoxia phase; the remaining time for each cycle was set as the re-oxygenation phase. Gas flow was regulated by timer-controlled solenoid valves and an O_2_ flow meter. During the hypoxia phase, the O_2_concentration in the chamber was rapidly decreased to 5% by adjusting the N2 flow rate. In contrast, the O_2_ concentration was increased to a maximum of 21% by rapidly flushing the chamber with compressed air. The chamber of animals in the SC group was continuously flushed with compressed air or any special treatment. Supplementary Table S1 provides specific details of the IH challenge.

To confirm hypoxia following IH exposure, the blood gas tension of 2 rats per group was measured. Each rat was anesthetized with 25% urethane (4 mL/kg), and arterial lines were surgically inserted into the right common carotid artery. The line was heparinized, and it exited the cage through a small aperture. We measured arterial blood gases at different time points in the hypoxia cycle after the rats had been allowed to adapt to the hypoxic conditions for at least 10 min. Minimum partial pressure of oxygen (PO_2_) and maximum partial pressure of carbon dioxide (PCO_2_) were measured in 3 consecutive hypoxia cycles. Supplementary Figure S1 shows changes of PO_2_ in response to chamber oxygen concentration. Analysis confirmed that oxygenation profiles in our rat model system mimicked those in patients with OSA.

### Animal anatomy and sample preparation

After 6 weeks of challenge, all animals were anesthetized with 3% pentobarbital (30 mg/kg), and an arterial blood sample was obtained from the right femoral artery. Serum was isolated and frozen at -80°C. Residual blood was cleaned, and animals were weighed. Next, small (1 cm × 0.5 cm) sections of tissue were excised from the right ventricle of the heart, snap frozen in liquid nitrogen, and stored at -80°C. On the day of analysis, frozen pericardium was thawed at 4°C and then at room temperature. Once fully thawed, samples were homogenized on ice. The homogenate was centrifuged (Sorvall Legend RT; Germany) at 4°C at 3000 *g* for 10 min, and the supernatant was saved for a future experiment.

### Enzyme-linked immunosorbent assay (ELISA)

Serum levels of tumor necrosis factor (TNF)-α, intracellular adhesion molecule (ICAM)-1, and interleukin (IL)-10 were measured using ELISA kits purchased from Ruike Biological Technology Company (China) and were performed according to the manufacturer’s protocol. Briefly, 50 μL of serum sample was mixed with 50 μL of assay diluent, then 100 µL of diluent alone (negative control) and 100 μL of serially diluted standards (positive control) were added. Solutions were added to a 96-well plate pre-coated with specific monoclonal antibodies to the antigen of interest and incubated at room temperature for 2 h. Following incubation, the plate was washed with 0.01 M phosphate buffered saline 3 times. Next, samples were incubated with appropriate biotinylated antibody and streptavidin-horseradish peroxidase conjugates. A colorimetric reaction was initiated by adding chromogen substrate and it was stopped by the addition of 1N H_2_SO_4_solution. Wells containing substrate alone and stop solution alone were included as controls. Absorbance was measured at 450 nm using a microplate reader (Labsystems Multiskan, USA). The mean readings of blank wells were subtracted from wells with sample to determine the final value. Standard curves were generated to calculate the concentration of each antigen.

### Western blot analysis

We performed western blot analysis to determine the levels of phosphorylated NF-κB p65 and HIF-1α in the harvested heart tissue. Nuclear fractions were obtained from myocardial tissues using cellular fractionation reagents from Beyotime Inc. (China). Protein concentrations were determined using a bicinchoninic acid (BCA) protein assay kit (Bomaide Inc., China). Protein markers were purchased from Fermentas (China). Myocardial cell nuclear extracts (10 μg of protein) were run on sodium dodecyl sulfate (SDS)-polyacrylamide gradient gels. Protein samples were separated by electrophoresis and transferred to nitrocellulose membranes. Membranes were blocked with 5% non-fat milk in tris-buffered saline for 1 h and then incubated with anti-phosphorylated NF-κB p65 or anti-HIF-1α rabbit polyclonal antibody (1:200 dilution; Santa Cruz Biotechnology; USA) overnight at 4°C. Membranes were washed with tris-buffered saline and then incubated with horseradish peroxidase-conjugated anti-rabbit IgG secondary antibody (Boster Inc., China) for 1 h at room temperature. The secondary antibody was diluted 1:5000 in blocking solution. After washing, membranes were incubated with LumiGLO detection solution (Jing-mei Bioscience, China) and exposed to film (Kodak, USA). Membranes were then stripped, blocked, and re-probed with antibodies to detect GAPDH, which was used as a loading control. Films were scanned, and the intensities of protein bands were quantified using Image J software (National Institutes of Health, USA). The protein expression levels were normalized to levels of GAPDH.

### Statistical analysis

Data are reported as means±SD. Statistical comparisons between different groups of rats were performed using a general linear model one-way ANOVA and *post hoc* Tukey’s test. A P value <0.05 was considered to be statistically significant. All statistical analyses were performed with IBM SPSS Statistics, Version 19.0 (IBM Corp., USA).

## Results

### Circulating cytokines

**Figure 1:**
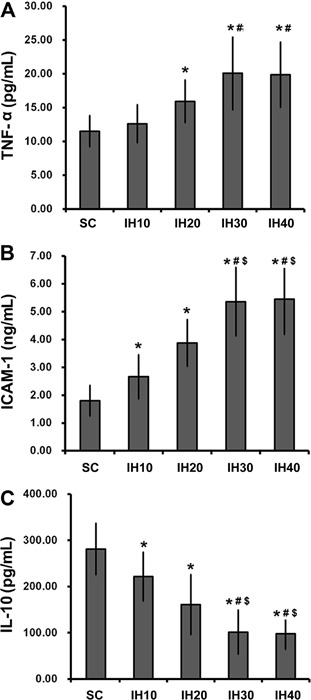
Serum levels of circulating cytokines in groups exposed to different intermittent hypoxia (IH) frequencies. Serum levels of TNF-α (*A*), ICAM-1 (*B*), and IL-10 (*C*) were measured in animals from each group (n=8 each). Data are reported as means±SD. Rats received compressed air (SC) or IH at a frequency of 10 (IH10), 20 (IH20), 30 (IH30), and 40 (IH40) times/h. TNF-α: tumor necrosis factor alpha; ICAM-1, intracellular adhesion molecule-1; IL-10, interleukin-10. *P<0.05 *vs* SC; #P<0.05 *vs* IH10; ^$^P<0.05 *vs* IH20 (one-way ANOVA and *post hoc* Tukey’s test).

Supplementary Table S2 summarizes the quantification of circulating cytokine levels. In general, serum levels of circulating cytokines were significantly altered in the four IH groups compared with either the SC group or the NC group (F=9.676, 27.318, 20.594; P<0.001). One exception was TNF-α, which was not significantly increased in the IH10 group (Figure 1). This suggested that the induction of a complete inflammatory response in this model required an IH frequency greater than 10 times/h. While serum levels of TNF-α (Figure 1A, only in groups IH20, IH30 and IH40) and ICAM-1 (Figure 1B) were increased by IH challenge (all IH groups), levels of IL-10 (Figure 1C) were decreased in all IH groups. Importantly, all changes in cytokine levels were dependent on IH frequency, with the greatest changes observed in groups with a higher frequency of challenge (IH20 *vs* IH30 and IH40). Taken together, these data support a relationship between systemic inflammation and IH.

### NF-κB phosphorylation and HIF-1α expression

**Figure 2:**
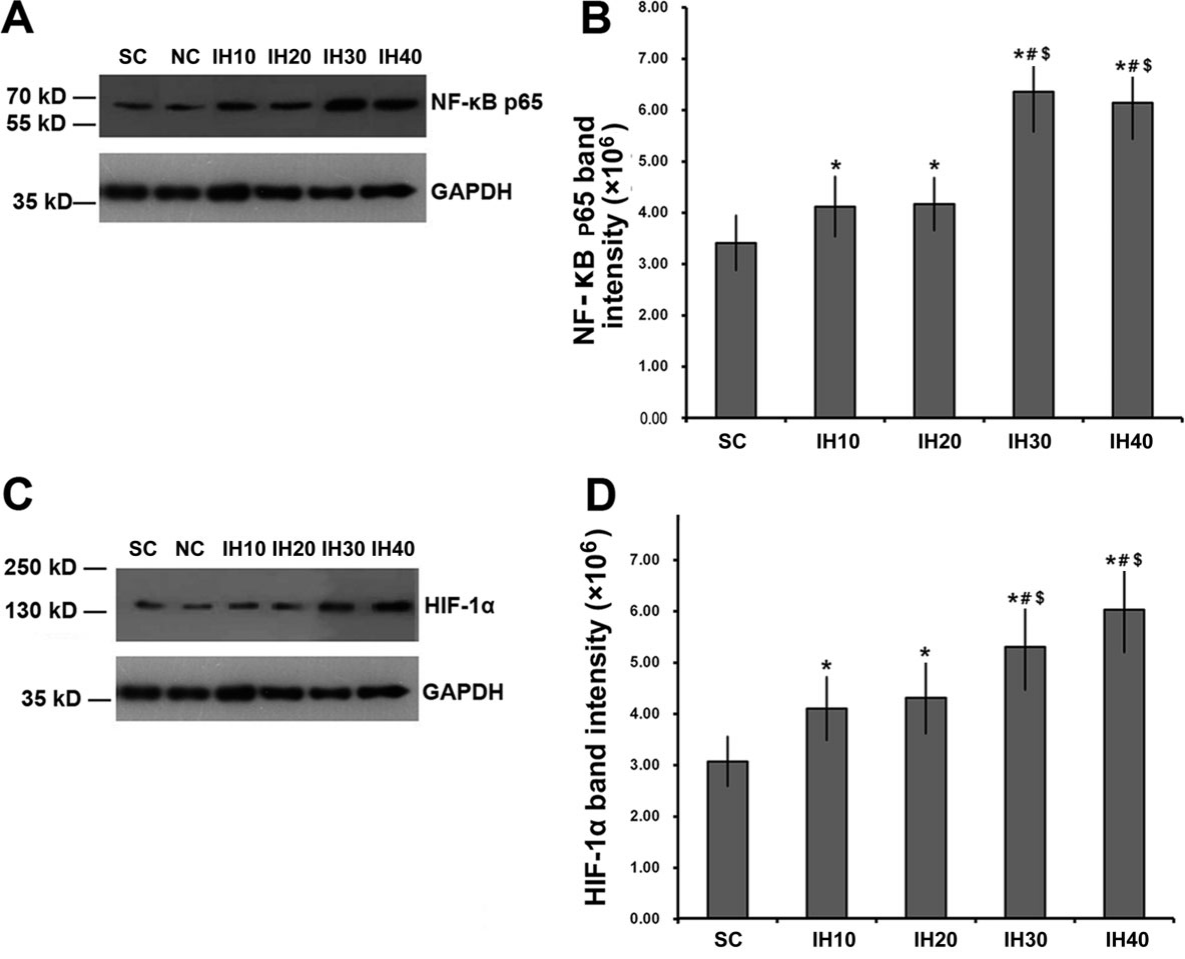
Changes in myocardial expression of phospho-nuclear factor kappa B (NF-κB) and hypoxia inducible factor-1α (HIF-1α) with increasing intermittent hypoxia (IH) frequency. *A*, Western blot analysis of phosphorylated NF-κB p65 in rats challenged with increasing frequencies of IH. GADPH was used as a loading control. *B*, The intensity of phosphorylated NF-κB p65 was normalized to that of GADPH, which was then plotted as means±SD. *C*, Western blot analysis of HIF-1α in rats challenged with increasing frequencies of IH. GADPH was used as a loading control. *D*, The intensity of HIF-1α was normalized to that of GADPH, which was then plotted as means±SD. *P<0.05 *vs* SC; ^#^P<0.05 *vs* IH10; ^$^P<0.05 *vs* IH20 (one-way ANOVA and *post hoc* Tukey’s test).

Supplementary Table S2 summarizes the quantification of tissue protein levels. Phosphorylated NF-κB p65 levels in nuclear extracts from myocardial tissues were significantly increased in IH rats compared with controls (Figure 2A and B; F=35.089; P<0.001). The increase in phospho-p65 was frequency-dependent, with more phosphorylated protein detected in rats receiving higher frequency IH (P<0.05 for IH10 or IH20 *vs* SC or NC; P<0.01 for IH30 or IH40 *vs* SC or NC). Importantly, there was no significant difference in the extent of phospho-p65 induced by IH10 *vs* IH20 or by IH30 *vs* IH40 (P>0.05).

We also examined changes in HIF-1α expression in each experimental group. Interestingly, changes in expression of HIF-1α mirrored the changes observed for phospho-p65. That is, HIF-1α levels were significantly increased in IH30 and IH40 rats compared with controls in a frequency-dependent manner (Figure 2C and D). Heart tissue from rats in groups IH10 and IH20 showed similar levels of HIF-1α. Tissue from animals in groups IH30 and IH40 were significantly increased compared with group IH10, while IH30 was significantly increased compared with group IH20 but there was no difference between IH10 and IH20.

### Tempol attenuated the inflammatory effects of intermittent hypoxia

**Figure 3:**
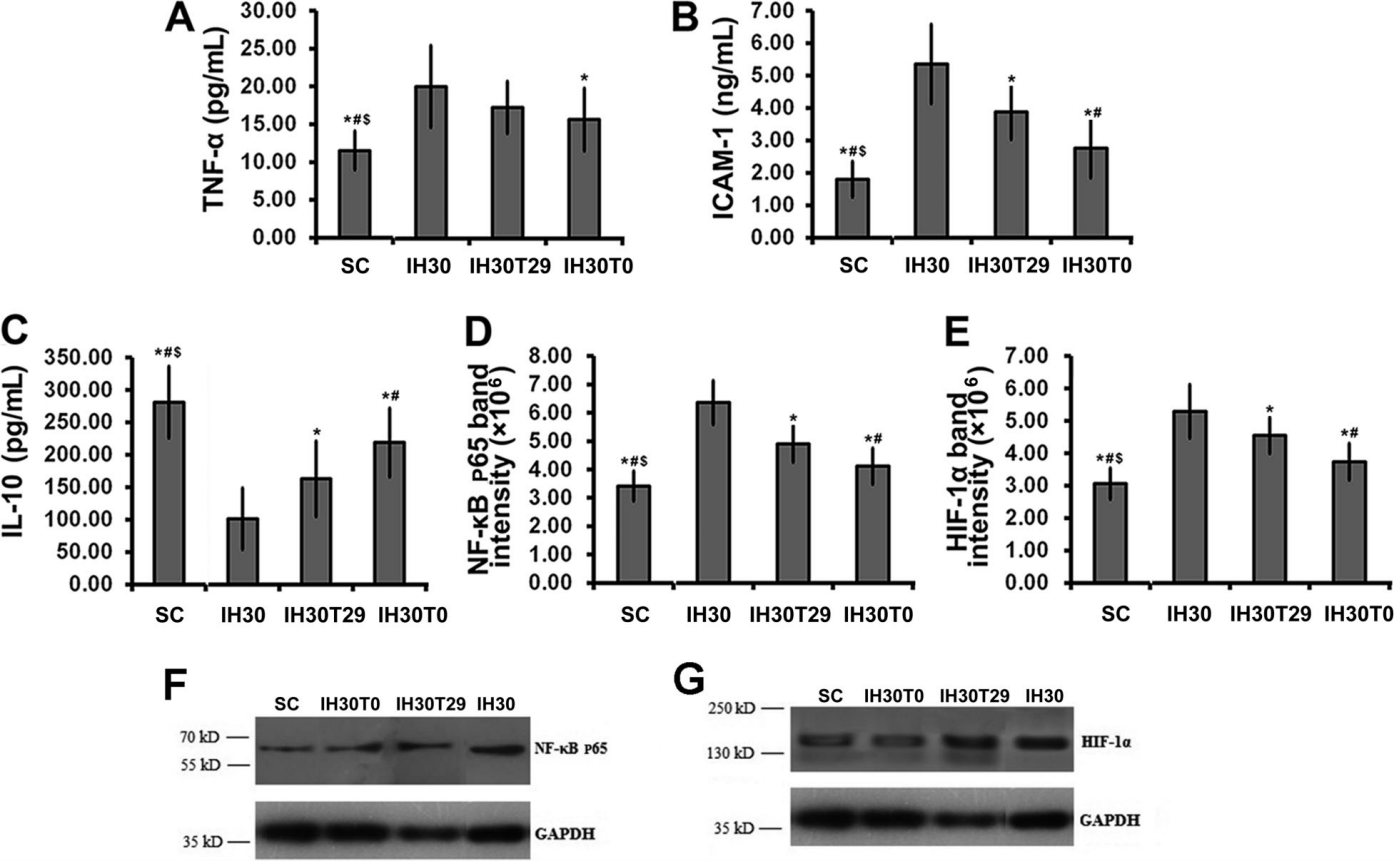
Changes in serum levels of circulating cytokines with tempol treatment. Animals received intermittent hypoxia (IH) 30 times/h as in the IH30 group. In addition, 1 mL/kg of 10% tempol was administered each time before IH exposure starting from the first day of treatment (IH30T0 group) or starting from day 29 (IH30T29 group) of the procedure. Serum levels of tumor necrosis factor (TNF)-α (*A*), intracellular adhesion molecule (ICAM)-1 (*B*), and interleukin (IL)-10 (*C*) were measured in the animals (n=8 per group) and compared with animals from the compressed air (SC) group. Data are reported as means±SD. *D*, Myocardial nuclear NF-κB phosphorylation in different IH30 groups with or without tempol intervention were determined. The quantitative data of *D* are reported as means±SD. *E*, Myocardial hypoxia inducible factor-1α (HIF-1α) protein levels in the IH30 groups with or without tempol intervention were determined. *F*, Representative Western blot for NF-κB phosphorylation. *G*, Representative Western blot for HIF-1α. *P<0.05 *vs* IH30; ^#^P<0.05 *vs* IH30T29; ^$^P<0.05 *vs* IH30T0 (one-way ANOVA and *post hoc* Tukey’s test).

We next determined whether tempol administration had an effect on IH-mediated inflammation and NF-κB activation. The potential effects of tempol were assessed by comparison against IH30 alone and against the normal oxygen (SC) control group. Quantitative data are summarized in Supplementary Table S3. When administered on day 0 (IH30T0), tempol attenuated the IH30-mediated increase in TNF-α (P<0.01; Figure 3A); however, the effect in the IH30T29 group was not significant. Consistent with this, the IH30-mediated increase in ICAM-1 was also attenuated by tempol administration (P<0.05; Figure 3B). Levels of IL-10 were decreased in the IH30 group, and this effect was abrogated by tempol whether treatment began on day 0 or on day 29 (Figure 3C).

Finally, we examined the effect of tempol treatment on myocardial NF-κB p65 and HIF-1α expression, which was significantly different between IH and control rats. The IH30-mediated increase of NF-κB p65 and HIF-1α was significantly attenuated in both the IH30T29 and IH30T0 groups (Figure 3D and E). Importantly, earlier tempol treatment (day 0) resulted in a greater benefit compared with treatment beginning on day 29. Taken together, the data showed that treatment with the antioxidant tempol had the ability to relieve or prevent inflammation resulting from IH.

## Discussion

In the present study, rats receiving IH with a high frequency displayed more severe inflammation than those receiving low-frequency IH. Moreover, levels of IL-10 were decreased by IH challenge. Our data indicated that IH activated myocardial NF-κB, resulting in systemic inflammation.

NF-κB is a transcription factor involved in cellular responses to various stimuli such as stress or infection. Its activity has been associated with atherosclerosis. Additionally, NF-κB regulates the expression of a number of genes involved in cell adhesion and inflammation including IL-6, TNF-α, E-selectin, vascular cell adhesion molecule-1, ICAM-1, and L-selectin. Importantly, all of these factors were elevated in patients with obstructive sleep apnea syndrome (OSAS) (7-12). Another transcription factor important for inflammatory responses is HIF-1α. Expression of this protein is increased under low oxygen concentrations because it helps both cellular and systemic responses to this type of stress. HIF-1α is transcriptionally regulated by NF-κB (13). Importantly, hypoxia and inflammation are tightly linked within the cell (14). The results of the present study showed that increased IH challenge increased HIF-1α levels. Because HIF-1α is necessary for adaptive changes of cells in response to hypoxia, the observation of increased HIF-1α suggested that the cells had adapted to improve survival and energy metabolism by inducing the transcription of a series of genes that participate in angiogenesis, iron metabolism, glucose metabolism, and cell proliferation/survival (15). Nevertheless, increased HIF-1α indicates that the cells are in a challenged state.

*In vitro* cell culture models showed that IH preferentially activates inflammatory pathways instead of the adaptive HIF-1 pathway (16). Moreover, clinical studies have shown that OSAS activates NF-κB, which is inhibited by continuous positive airway pressure therapy (17,18). Other studies have established that NF-κB activity is increased in myocardial (17) and liver (19) tissues in mice exposed to IH. One important study showed that the level of NF-κB P65 expression in aortic endothelial cells of rats exposed to IH was significantly higher than in rats exposed to either normal oxygen or to continuous hypoxia. Moreover, the same study showed that cellular stress worsened with increasing levels of IH (20). This provided evidence that the activation of NF-κB, to a certain extent, is dependent on the degree of IH. These studies also support the fact that IH activates NF-κB, which subsequently promotes the expression of inflammatory mediators and adhesion molecules.

Our data showed that the IH-mediated increase in inflammation and NF-κB was dependent upon the frequency of IH. However, because there was no significant difference between groups IH30 and IH40, there is likely a maximum limit of inflammation and NF-κB activity that can be induced under these conditions. This might be explained by three possibilities. First, with increasing intermittent hypoxic frequency, the period of re-oxygenation is shortened and, at some threshold, this likely results in a situation that is similar to a state of continuous hypoxia. Under these conditions, the adaptive HIF-1 pathway may dominate over NF-κB activation, a finding supported by the work of Yuan et al. (21). Second, high frequency IH may trigger compensatory mechanisms to protect against excessive inflammatory injuries. NF-κB signaling is controlled by a negative feedback loop to limit both acute and excessive inflammation. Finally, we cannot rule out the possibility that a higher frequency of IH (greater than 40 times/h) or IH administered over a long period (longer than 6 weeks) would result in further increases in inflammation and NF-κB activity. However, the answer to this question would require additional research.

In this study, we observed that IH challenge of rats lead to decreased expression of circulating IL-10, a critical anti-inflammatory mediator (22), and increased TNF-α and ICAM-1, two molecules involved in inflammatory processes. IL-10 inhibits the nuclear translocation of NF-κB and subsequently decreases levels of TNF-α, IL-6, and C-reactive protein, which are all pro-inflammatory mediators (23). Furthermore, previous studies have shown that IL-10 is decreased in OSA patients (23-25). However, TNF-α activates NF-κB and MAPK pathways, which have important roles in inflammation and apoptosis (26) and ICAM-1 is involved in the recruitment of macrophages and granulocytes, which may increase the local inflammatory load (27). Thus, our results are in agreement with these studies. This impaired inflammatory response could explain why patients with OSA have a greater risk of cardiovascular disease compared with the general population. However, this is likely a complex biological process, because IL-10 produced by T lymphocytes was not associated with OSA severity (28). Despite this, our data support the concept that inflammation is a key mediator of IH-induced injury in patients with OSA.

Anti-inflammatory and anti-oxidant therapies are promising options to decrease oxidative stress and inhibit cardiovascular inflammation. A previous study showed that the exogenous administration of the antioxidant N-acetyl-L-cysteine significantly improved IH-mediated myocardial injury in a mouse model (29). Consistent with this, we found that treatment with tempol alleviated some of the IH-induced inflammation in our rat model. Tempol is a nitrogen monoxide molecule with antioxidant properties and has previously been used to successfully treat IH-induced injuries. For example, tempol was shown to improve IH-mediated skeletal muscle injuries in rats (30). Furthermore, Troncoso et al. (31) showed that tempol decreased the blood pressure of rats exposed to IH for 14 days, an effect likely mediated by a decrease in serum levels of endothelin-1. These data support the effective use of tempol to combat IH-induced injuries.

Here, we showed that tempol treatment significantly attenuated IH-mediated increases in TNF-α, ICAM-1, and NF-κB. This is consistent with an anti-inflammatory effect of tempol in this rat model. Additionally, we found that early tempol intervention was more beneficial than tempol treatment started at a later time point. Additional studies are required to determine the effects of longer tempol exposure in this model. In addition, we only superficially explored the effects of IH on the complex inflammation processes. In depth analyses of inflammation are necessary to improve our understanding of the effects of IH on inflammation.

In conclusion, IH caused both systemic and local cardiovascular inflammation. IH not only increased NF-κB activity, HIF-1α and inflammation, but also weakened the anti-inflammatory response, thus upsetting the overall balance of this biological pathway. This could be a major underlying problem in patients with OSA. Moreover, treatment with antioxidants, such as tempol, may be therapeutically beneficial for the treatment or prevention of OSAS-related cardiovascular disease via the inhibition of ROS and a subsequent decrease in the inflammatory response.

## Supplementary Material


