# Supplementary material for: Inflammation induced by increased frequency of intermittent hypoxia is attenuated by tempol administration
Source: Braz J Med Biol Res. 2015 Sep 18;48(12):1115–21. doi: 10.1590/1414-431X20154487 (PMC4661028; doi:10.1590/1414-431X20154487)
Supplement: Supplementary file 1 [file 1414-431X-bjmbr-48-12-01115-supp4487.pdf]

Figure S1. Changes in oxygen concentration (lower tracing) in the hypoxia chamber, and partial pressure of oxygen (PaO_2, upper tracing) in experimental animals responding to hypoxia conditioning.

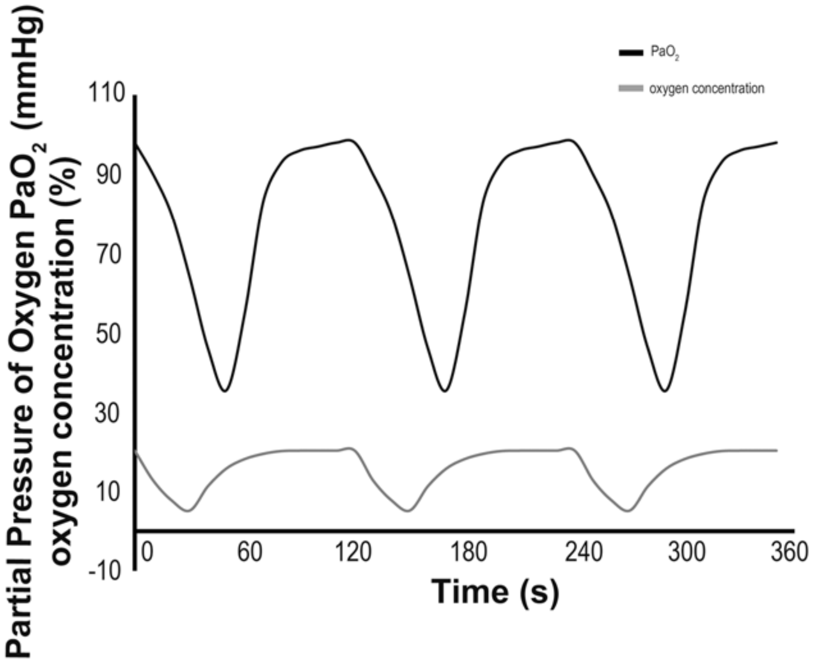

Table S1. Intermittent hypoxia treatment.

Group	Sample size	Frequency (times/h)	Hypoxia time (s)	Re-oxygen time (s)	Minimum oxygen concentration (%)	Total exposure time (weeks)
IH10	8	10	30 s	330 s	5%	6
IH20	8	20	30 s	150 s	5%	6
IH30	8	30	30 s	90 s	5%	6
IH40	8	40	30 s	60 s	5%	6
IH30T0[a]	8	30	30 s	90 s	5%	6
IH30T29[a]	8	30	30 s	90 s	5%	6
SC	8	—	—	sustained normal oxygen	sustained normal oxygen	6
NC	8	—	—	—	—	—

Four groups were exposed to intermittent hypoxia (IH) 10, 20, 30, or 40 times/h. [a]Two other experimental groups were challenged with IH (30 times/h) plus tempol (T), either beginning on day 0 (IH30T0) or on day 29 (IH30T29) to determine whether tempol protected against IH damage. SC: compressed air; NC: normal air.

Table S2. Levels of serum cytokines and tissue proteins in animals of intermittent hypoxia (IH) and control groups.

	SC	IH10	IH20	IH30	IH40
TNF-α (pg/mL)	11.52 ± 2.55	12.62 ± 2.81	15.93 ± 3.17*	20.10 ± 5.42*#	19.87 ± 4.84*#
ICAM-1 (ng/mL)	1.80 ± 0.55	2.66 ± 0.79*	3.88 ± 0.84*	5.36 ± 1.23*#$	5.45 ± 1.27*#$
IL-10 (pg/mL)	280.94 ± 55.36	221.40 ± 52.52*	160.79 ± 64.91*	101.31 ± 47.55*#$	97.85 ± 33.34*#$
NF-κB P65 (arbitrary units)	3.41 ± 0.53	4.12 ± 0.58*	4.17 ± 0.51*	6.36 ± 0.78*#$	6.14 ± 0.70*#$
HIF-1α (arbitrary units)	3.06 ± 0.48	4.09 ± 0.61*	4.30 ± 0.69*	5.29 ± 0.83*#$	6.02 ± 0.83*#$

Data are reported as means±SD. Animals were exposed to intermittent hypoxia (IH) 10, 20, 30, or 40 times/h or compressed air (SC) (n=8 animals/group). TNF-α: tumor necrosis factor; ICAM-1: intracellular adhesion molecule-1; IL-10: interleukin-10; NF-κB: nuclear factor kappa B; HIF-1α: hypoxia inducible factor-1α. *P<0.05 *vs* SC; #P<0.05 *vs* IH10; $P<0.05 *vs* IH20 (one-way ANOVA and *post hoc* Tukey's test).

Table S3. Levels of serum cytokines and tissue proteins in tempol-treated animals compared to control and IH30 groups.

	SC	IH30	IH30T29	IH30T0
TNF-α (pg/mL)	11.52 ± 2.55*#$	20.10 ± 5.42	17.32 ± 3.45	15.72 ± 4.16*
ICAM-1 (ng/mL)	1.80 ± 0.55*#$	5.36 ± 1.23	3.88 ± 0.86*	2.76 ± 0.92*#
IL-10 (pg/mL)	280.94 ± 55.36*#$	101.31 ± 47.55	162.93 ± 58.37*	219.04 ± 53.15*#
NF-κB P65 (arbitrary units)	3.41 ± 0.53*#$	6.36 ± 0.78	4.90 ± 0.65*	4.12 ± 0.64*#
HIF-1α (arbitrary units)	3.06 ± 0.48*#$	5.29 ± 0.83	4.55 ± 0.56*	3.74 ± 0.57*#

Data are reported as means±SD. Animals were exposed to intermittent hypoxia (IH) 30 times/h, with or without tempol (T) at 0 or 29 days, or compressed air (SC) (n=8 animals/group). TNF-α: tumor necrosis factor; ICAM-1: intracellular adhesion molecule-1; IL-10: interleukin-10; NF-κB: nuclear factor kappa B; HIF-1α: hypoxia inducible factor-1α. *P<0.05 *vs* IH30; #P<0.05 *vs* IH30T29; $P<0.05 *vs* IH30T0 (one-way ANOVA and *post hoc* Tukey's test).
